# Seroprevalence study for selected zoonotic vector-borne pathogens in sheep from endemic areas of Croatia

**DOI:** 10.3389/fvets.2025.1602706

**Published:** 2025-06-02

**Authors:** Ljubo Barbic, Vladimir Stevanovic, Maja Mauric Maljkovic, Gorana Miletic, Ivona Coric, Vladimir Savic, Viktor Masovic, Maja Bogdanic, Alan Medic, Tatjana Vilibic-Cavlek

**Affiliations:** ^1^Department of Microbiology and Infectious Diseases with Clinic, Faculty of Veterinary Medicine, University of Zagreb, Zagreb, Croatia; ^2^Department of Animal Breeding and Livestock Production, Faculty of Veterinary Medicine, University of Zagreb, Zagreb, Croatia; ^3^Poultry Center, Croatian Veterinary Institute, Zagreb, Croatia; ^4^Department of Virology, Croatian Institute of Public Health, Zagreb, Croatia; ^5^School of Medicine, University of Zagreb, Zagreb, Croatia; ^6^Department of Epidemiology, Zadar County Institute of Public Health, Zadar, Croatia

**Keywords:** tick-borne encephalitis virus, *Borrelia burgdorferi* s.l., West Nile virus, Usutu virus, Crimean-Congo hemorrhagic fever virus, sentinels

## Abstract

Surveillance is crucial in controlling and preventing vector-borne zoonotic diseases (VBDs). We analyzed the seroprevalence of selected vector-borne zoonotic pathogens in sheep from endemic areas and their role as possible sentinels for VBDs. A total of 300 sheep from seven farms at three micro-locations were tested for the presence of IgG antibodies against tick-borne encephalitis virus (TBEV), West Nile virus (WNV), Usutu virus (USUV), *Borrelia burgdorferi* s.l., and Crimean-Congo hemorrhagic fever virus (CCHFV) using ELISA with confirmation of borderline/positive results by VNT. Seropositivity for at least one pathogen was observed in 18.0% (54/300) of sheep. The highest seroprevalence was confirmed for TBEV (9.7%; 29/300), followed by WNV (3.0%; 9/300) and *B. burgdorferi* s.l. (2.7%; 8/300), while USUV and inconclusive flavivirus (TBEV/WNV/USUV) infections had the same seroprevalence of 1.3% (4/300). None of the serum samples tested positive for CCHFV. Geographic micro-location was a significant risk factor for USUV (*p* = 0.045), TBEV (*p* = 0.03), and *B. burgdorferi* s.l. (*p* = 0.015) infections, but not for WNV. The farm distance from the household (TBEV *p* < 0.001, *B. burgdorferi* s.l. *p* = 0.005) and sheep breed (TBEV *p* < 0.001, *B. burgdorferi* s.l. *p* < 0.001) were found as risk factors for seropositivity to tick-borne (TBEV, *B. burgdorferi* s.l.), but not to mosquito-borne diseases (WNV, USUV). Of the other risk factors, sheep shearing was statistically significant, with unshared sheep showing a higher probability of tick-borne diseases (*p* = 0.048). Sex, age, herd size, and the presence of clinical signs were not associated with the seroprevalence. Serologic evidence of VBDs suggests their sentinel potential for mapping micro-foci of zoonotic pathogens’ activity and identifying high-risk areas for public health. Further studies are needed to confirm this observation.

## 1 Introduction

Zoonotic vector-borne diseases (VBDs) are becoming an increasing public health problem in many regions of the world (1). VBDs represent more than 17% of all infectious diseases, causing more than 700,000 human deaths annually, with the highest disease burden in tropical and subtropical areas. The spread of VBDs has been facilitated by different factors, including global travel and trade, unplanned urbanization, climate change, and the vectors’ adaptation and spread (2).

Among vector-borne pathogens, *Borrelia burgdorferi* s.l. (Lyme disease; LD) and tick-borne encephalitis virus (TBEV) are most widely distributed in Europe (1). Other flaviviruses, such as West Nile virus (WNV) and Usutu virus (USUV), are also endemic in many European countries, causing outbreaks (WNV) or sporadic infections (USUV) in humans (3). In addition, several epidemics of Crimean-Congo hemorrhagic fever (CCHF) have occurred in EU/EEA neighboring countries since 2013, including the Balkan region, Russia, and Turkey (4).

Since zoonotic VBDs are spreading rapidly, surveillance is crucial in their prevention and control. To define priorities for developing integrated surveillance systems that accurately model and predict the human risk of VBDs, it is important to understand the practical options of connecting the surveillance data of both animals and humans (5). As clinical cases of emerging diseases in humans usually indicate a widespread of zoonotic pathogens in an area, various animal surveillance models are used as an early warning system, yielding valuable results. Animal surveillance can address the significant public health challenge of gathering information on the introduction and emergence of new pathogens in a given area, a prerequisite for an effective response to protect human health (6–8).

Different animal species have been tested as sentinels for VBDs, including wild birds (WNV and USUV) (9), horses (TBEV, WNV, and USUV) (10), poultry (WNV and USUV) (11), and dogs (TBEV and WNV) (12). Captive and free-ranging birds have been used for WNV surveillance for decades. As primary WNV reservoirs, infections in birds occurred more frequently than in humans and horses (13). In addition, chickens (*Gallus gallus domesticus*) have been routinely used for arbovirus surveillance and monitoring in different settings and on different continents. After infection, chickens do not exhibit any clinical signs but produce neutralizing antibodies (11). The use of sentinel chickens seems to be a more sensitive indicator of virus activity when compared with the detection of seroconversion in wild birds (14). While clinically apparent WNV infections in horses are rarely observed, seroprevalence studies in horses may allow the tracing of flavivirus transmission and help to estimate the risk for human infections (10). Whereas some studies provided evidence that dogs could be useful sentinels for WNV monitoring (15), others indicated that the role of dogs and horses in the early detection of human cases is debatable (16).

However, studies that use sheep as sentinels to predict human risk are limited. A study conducted in Germany has shown that seroprevalence in free-ranging animals, particularly in sheep and goats, can be a useful additional tool to identify TBEV foci in both endemic and non-endemic areas (17). Very few studies have analyzed WNV infection in sheep, with no data on USUV (18). Sheep and cattle became infected with the CCHFV in experimental inoculations but developed only mild and transient fever. The viremia duration and level are usually low, with detectable antibodies shortly after cessation of viremia (19). Although CCHF in domestic ruminants is typically subclinical, there is some evidence that they become reservoirs and can be used as sentinels for the circulation of CCHFV, especially in non-endemic areas (20).

In Croatia, *B. burgdorferi* s.l., TBEV, WNV, and USUV are the most commonly detected vector-borne zoonotic pathogens, all endemic in continental regions (21). The reported number of LD varies from 400 to 800. In addition, TBE is continuously recorded with a bimodal seasonality (April–August and October–November). The LD and TBE endemicity is highest in northwestern and eastern counties between the Sava and Drava rivers (21, 22). WNV infections in humans, horses, and poultry have been continuously reported in Croatia since 2012 (21), while USUV infections were detected sporadically in humans (2013, 2018, 2024) and birds (2018, 2022) (23–25). CCHFV has not been detected in Croatia so far.

The multidisciplinary approach enables the early detection of an increase in pathogen activity of VBDs or confirmation of the emergence of a new pathogen in a given area, as has also been observed in Croatia in recent years (6, 7). Chickens and horses were used as sentinels to detect seasonal WNV incursions in Croatia, revealing a significant correlation between the geographical distribution of high WNV seroprevalence in tested animals and human WNV infections (26). However, data on the sheep are limited.

This study aimed to analyze the seroprevalence of selected vector-borne zoonotic pathogens in sheep from endemic areas in eastern Croatia and assess the potential role of sheep as sentinels of VBDs.

## 2 Materials and methods

**Figure 1:**
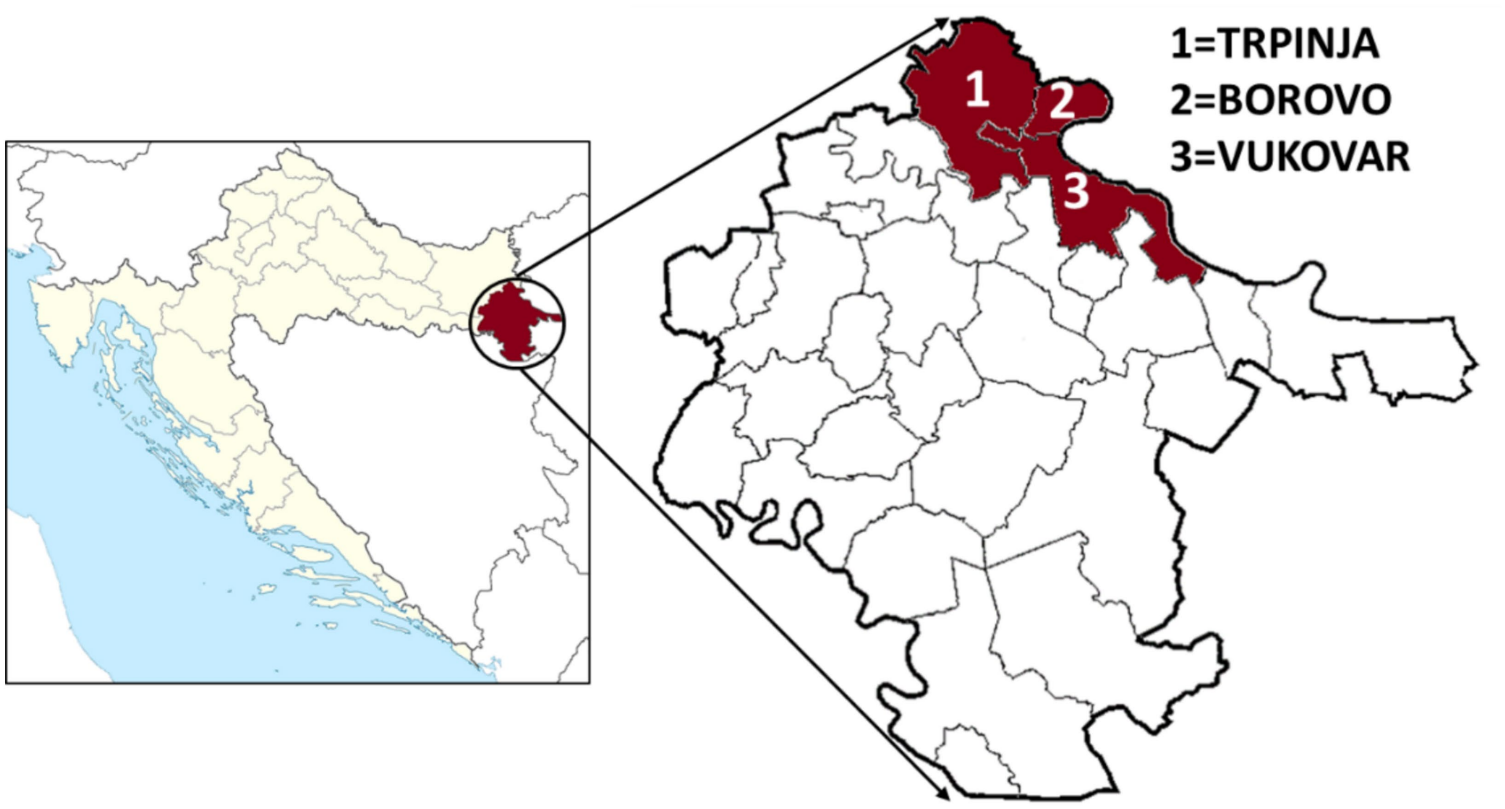
Sheep sampling area in Croatia (Vukovar-Srijem County) with three micro-locations.

In this study, 300 sheep from Vukovar-Srijem County, the easternmost region of Croatia, were tested for the presence of IgG antibodies against TBEV, WNV, USUV, *B. burgdorferi* s.l., and CCHFV. Blood samples were collected from animals from seven farms at three micro-locations: four in Borovo, one in Vukovar, and two in Trpinja (Figure 1).

Sample size calculations were performed using the RiBESS+ tool developed by the European Food Safety Authority (27). The calculation incorporated the official sheep population data for Vukovar-Srijem County, the reported sensitivity of the enzyme-linked immunoassay (ELISA) used, and an assumed seroprevalence of 1%, reflecting the absence of previously documented USUV infections in sheep.

Serum samples were collected in the second half of April to obtain reliable data of the epidemiological status before the onset of peak vector activity.

Epidemiological and clinical data of the tested sheep are presented in Table 1. Clinical signs were assessed both at the time of sampling and retrospectively based on owner-reported observations over the preceding 12 months. The following categories were evaluated: neurological, respiratory, reproductive, gastrointestinal, dermatological signs, and lameness. Information regarding any additional observed clinical signs was also collected. Clinical examinations performed during sampling across all seven farms revealed no detectable clinical abnormalities. However, according to the owner of Farm 1, located in Borovo, episodes of dermatitis affecting all animals were reported during the winter (approximately 3 months prior), characterized by skin erythema and partial wool loss. These lesions resolved spontaneously without the administration of any treatment.

**Table 1:** Epidemiological data on sheep farms from Vukovar-Srijem County included in the study.

Characteristic	Vukovar	Trpinja		Borovo			
	Farm 1	Farm 1	Farm 2	Farm 1	Farm 2	Farm 3	Farm 4
Flock size	100	286	50	50	10	150	70
% of flock tested	73.0	28.0	40.0	64.0	40.0	33.3	57.1
Tested females/males	73/0	79/1	20/0	32/1	4/0	47/3	38/2
Breed	Romanov	Merino	Merino	Tsigai	Merino	Tsigai	Romanov
Average age and range (years)	3.8 (1.1–4.8)	4.4 (1.4–12.3)	6.2 (2.1–9.2)	4.1 (1.1–8.2)	6.2 (5.6–6.6)	4.0 (2.2–4.9)	3.5 (2.3–5.0)
Observed clinical signs	No	No	No	Yes	No	No	No
Water body type	Stream	River	Stream	River	River	River	River
Distance to water (km)	0.02	10.0	0.2	0.1	0.2	0.1	0.3
Distance to households (km)	0.5	0.05	0.05	2.0	0.05	1.5	0.02
Shearing performed	No	Yes	Yes	No	No	No	No

All serum samples were screened using an ELISA for the detection of flavivirus antibodies (WNV, USUV, and TBEV) with the ID Screen^®^ Flavivirus Competition (Innovative Diagnostics, Grabels, France). Samples with borderline or positive ELISA results were additionally tested by a virus neutralization test (VNT), as described by Ilic et al. (28), to exclude cross-reactivity and confirm infection with a specific flavivirus. A VNT titer of ≥10 was considered a positive result. In the case of positive VNT results with more than one flavivirus, the pathogen with an antibody titer at least fourfold higher than the others was considered to be the causative agent. Samples with less than fourfold titer differences using different pathogens in VNT were considered inconclusive, classified as flavivirus-positive, and excluded from further risk factor analysis for a particular disease or group of diseases.

The ID Screen^®^ Borreliosis Double Antigen Multi-species ELISA (Innovative Diagnostics, Grabels, France) was used for the detection of antibodies against *B. burgdorferi* s.l., and the ID Screen^®^ CCHF Double Antigen Multi-species ELISA (Innovative Diagnostics, Grabels, France) for CCHFV antibodies.

All commercial ELISA assays were performed according to the manufacturer’s instructions.

Epidemiological data were systematically collected for each animal to assess possible risk factors associated with arboviral infections. The analyzed parameters included farm location, flock size, breed, sex, age, clinical signs, type of water bodies, and distance to water bodies and households. Risk factor analyses were conducted for each VBD, with additional analyses for tick-borne infections (TBEV, CCHFV, and *B. burgdorferi* s.l.) and mosquito-borne infections (WNV and USUV) due to epidemiological differences.

Statistical analyses were performed using Statistica v.14 (TIBCO Software Inc., 2020), Medcalc Odds Ratio Calculator v.23 (MedCalc Software Ltd., 2025), and R 4.4.0 (R Core Team, Vienna, Austria, 2024). Descriptive statistics are presented as numbers and percentages. Odds ratios (OR) with 95% confidence intervals (95% CI) were used for single bivariate risk factors. Logistic regression analysis was used to calculate ORs for numerical and multivariate risk factors, while ANOVA type II was used to assess the overall influence of the whole variable. When complete separation occurred for multivariate variables (cells with 0), Firth correction was applied using the logistf package. The relation between variables was analyzed using Pearson’s correlation coefficient (*r*; numerical variables) and Cramér’s *V* (*V*; nominal variables). Differences were considered significant at *p* < 0.05.

## 3 Results

Out of 300 tested sheep serum samples, 54 (18.0%) were positive for at least one vector-borne zoonotic pathogen (Table 2). The highest seroprevalence was confirmed for TBEV (9.7%). The seroprevalence of WNV was 3.0% *B. burgdorferi* s.l. 2.7%, while USUV and inconclusive flavivirus (TBEV/WNV/USUV) infections had the same seroprevalence of 1.3%. None of the serum samples tested positive for CCHFV.

**Table 2:** Seroprevalence of sheep to vector-borne zoonoses by micro-location and farm in Croatia (*N*, %).

Location	Farm	Number of tested animals	WNV	USUV	TBEV	Flavivirus	*B. burgdorferi* s.l.	Mosquito-borne pathogens	Tick-borne pathogens	Positive to at least one pathogen
Vukovar	Total	73	1 (1.4)	3 (4.1)	5 (6.9)	0 (0.0)	0 (0.0)	4 (5.5)	5 (6.8)	9 (12.3)
Trpinja	Farm 1	80	3 (3.8)	1 (1.3)	4 (5.0)	1 (1.3)	1 (1.3)	4 (5.0)^*^	5 (6.3)^*^	10 (12.5)
	Farm 2	20	0 (0.0)	0 (0.0)	1 (5.0)	1 (5.0)	0 (0.0)	0 (0.0)^*^	1 (5.0)^*^	2 (10.0)
	Total	100	3 (3.0)	1 (1.0)	5 (5.0)	2 (2.0)	1 (1.0)	4 (4.0)	6 (6.0)	12 (12.0)
Borovo	Farm 1	33	1 (3.0)	0 (0.0)	6 (18.2)	1 (3.0)	2 (6.1)	1 (3.0)^*^	8 (24.2)^*^	10 (30.3)
	Farm 2	4	2 (NA)	0 (0.0)	0 (0.0)	0 (0.0)	0 (0.0)	2 (NA)	0 (0.0)	2 (NA)
	Farm 3	50	2 (4.0)	0 (0.0)	11 (22.0)	1 (2.0)	5 (10.0)	2 (4.0)^*^	16 (32.0)^*^	19 (38.0)
	Farm 4	40	0 (0.0)	0 (0.0)	2 (5.0)	0 (0.0)	0 (0.0)	0 (0.0)	2 (5.0)	2 (5.0)
	Total	127	5 (3.9)	0 (0.0)	19 (15.0)	2 (1.6)	7 (5.5)	5 (3.9)	26 (20.5)	33 (26.0)
Total		300	9 (3.0)	4 (1.3)	29 (9.7)	4 (1.3)	8 (2.7)	13 (4.3)	37 (12.3)	54 (18.0)

All samples tested negative to Crimean-Congo hemorrhagic fever virus. *Numbers of positive animals without four flavivirus (TBEV/WNV/USUV)-positive animals, in which it was not possible to determine the pathogen; NA—insufficient sample size of tested animals for farm-level analysis; WNV, West Nile virus; USUV, Usutu virus; TBEV, tick-borne encephalitis virus; mosquito-borne pathogens: WNV, USUV; tick-borne pathogens: TBEV, *B. burgdorferi* s.l.

TBEV seropositivity was confirmed at all micro-locations in six out of seven farms, with seroprevalence rates from 5.0 to 15.0%. WNV-positive animals were confirmed at all micro-locations on five of the seven farms, with seroprevalence ranging from 1.4 to 3.9%. The lowest seroprevalence was found for USUV, with only four positive animals confirmed on two farms at two micro-locations, with a seroprevalence of 4.1 and 1.0%, respectively (Table 2). Four samples (1.3%) were considered as flavivirus-positive due to cross-reactivity. Cross-reactivity between WNV and TBEV was confirmed in three samples and between USUV and TBEV in one sample (Table 2). Serological evidence of *B. burgdorferi* s.l. infection was confirmed in 8 animals (2.7%) at two micro-locations with seroprevalence rates of 1.0 and 5.5%, respectively.

Location as a risk factor was significant for USUV, TBEV and *B. burgdorferi* s.l. infections, but not for WNV. For mosquito-borne diseases, location had no significant influence, while it was found as a risk factor for tick-borne diseases (ANOVA *p* = 0.006) (Table 3).

**Table 3:** Seroprevalence of vector-borne zoonoses in sheep according to geographic and environmental risk factors in Croatia (OR, 95% CI, *p*).

Risk factor		WNV	USUV	TBEV	*B. burgdorferi* s.l.	Mosquito-borne pathogens	Tick-borne pathogens
Location	Borovo^*^	*p* = 0.54	***p* = 0.045**	***p* = 0.03**	***p* = 0.015**	*p* = 0.878	***p* = 0.006**
	Trpinja	0.76 (0.15–3.17) *p* = 0.71	3.86 (0.2–566.03) *p* = 0.37	**0.3 (0.1–0.78) *p* = 0.02**	0.24 (0.03–1.13) *p* = 0.07	1.02 (0.25–3.96) *p* = 0.98	**0.29 (0.1–0.7) *p* = 0.01**
	Vukovar	0.33 (0.02–2.12) *p* = 0.32	**12.46 (1.18–1684.65) *p* = 0.03**	0.41 (0.13–1.08) *p* = 0.09	**0.11 (0.001–0.92) *p* = 0.04**	1.39 (0.33–5.43) *p* = 0.63	**0.33 (0.11–0.84) *p* = 0.03**
Water bodies		0.27 (0.03–2.18) *p* = 0.22	6.84 (0.7–66.69) *p* = 0.098	0.55 (0.22–1.4) *p* = 0.21	0.13 (0.01–2.2) *p* = 0.16	0.98 (0.3–3.28) *p* = 0.98	0.44 (0.18–1.1) *p* = 0.08
Distance from water bodies (km)		1.034 (0.88–1.19) *p* = 0.65	0.99 (0.72–1.22) *p* = 0.91	0.91 (0.80–1.01) *p* = 0.104	0.91 (0.67–1.08) *p* = 0.37	1.02 (0.89–1.15) *p* = 0.75	0.92 (0.82–1.01) *p* = 0.097
Distance from household (km)		1.07 (0.38–2.55) *p* = 0.89	0.58 (0.05–2.46) *p* = 0.54	**2.50 (1.52–4.18) *p* < 0.001**	**4.50 (1.73–15.22) *p* = 0.005**	0.90 (0.36–1.94) *p* = 0.81	**2.74 (1.71–4.45) *p* < 0.001**

^*^Reference category in the logistic regression; the ANOVA *p*-value represents the influence of location; WNV, West Nile virus; USUV, Usutu virus; TBEV, tick-borne encephalitis virus; mosquito-borne pathogens: WNV/USUV; tick-borne pathogens: TBEV/*B. burgdorferi* s.l. Statistically significant differences are bold.

When analyzing the risk between micro-locations, Borovo was selected as a reference category, as the highest number of animals was tested there. No significant association between micro-location and WNV or USUV seropositivity was found at the location Trpinja, as was the case for a group of tick-borne diseases. In contrast, on the same location, the probability of TBEV infection (*p* = 0.02) and tick-borne diseases overall (*p* = 0.01) was significantly lower and a borderline reduction in the risk of *B. burgdorferi* s.l. infection was also observed (*p* = 0.07). In contrast, in Vukovar, we observed a significant increase in the risk of USUV infections (*p* = 0.03), a decrease in *B. burgdorferi* s.l. seropositivity (*p* = 0.04) as well as lower risk of tick-borne diseases (*p* = 0.03) (Table 3).

The type of water body and the distance of the farm from it as risk factors had no significant influence on the seroprevalence of any VBDs or a group of diseases (Table 3).

The distance of the farm from the household was found as an important risk factor for seropositivity to TBEV (*p* < 0.001), *B. burgdorferi* s.l. (*p* = 0.005) and accordingly also for tick-borne diseases (*p* < 0.001), but not for WNV, USUV, and mosquito-borne infections (Table 3). To exclude the influence of location on these results because of just one farm analyzed at Vukovar micro-location and two at Trpinja, the same findings were analyzed for the four farms in Borovo micro-location, and distance to households was confirmed as a significant risk factor also on the same location at the farm level (*p* = 0.01).

When analyzing differences in VBDs seroprevalence at the farm level, a significant impact on seroprevalence was confirmed (*p* = 0.002), but as only animals from one farm at the Vukovar micro-location were tested, a close association between farm and location (*V* = 1) was confirmed, so we do not present the results.

The sheep breed was confirmed as an important risk factor with a significantly higher probability of infections with TBEV (*p* = 0.003), *B. burgdorferi* s.l. (*p* = 0.002) and tick-borne pathogens (*p* < 0.001) in Tsigai sheep than in Romanov sheep, which were used as the reference category. The seroprevalence rates in Merinolandschaf sheep were not significantly different (Table 4). When analyzing the correlation between the variables, sheep breed and location, the correlation was high (*V* = 0.83, 95% CI 0.79–0.86), with only Merinolandschaf present in Trpinja and only Romanov sheep in Vukovar.

**Table 4:** Seroprevalence of vector-borne zoonoses in sheep according to host- and management-related risk factors in Croatia (OR, 95% CI, *p*).

Risk factor	WNV	USUV	TBEV	*B. burgdorferi* s.l.	Mosquito-borne pathogens	Tick-borne pathogens
Romanov breed*	*p* = 0.17	*p* = 0.18	***p* < 0.001**	***p* < 0.001**	*p* = 0.672	***p* < 0.001**
Merino breed	5.77 (0.91–111.62) *p* = 0.11	0.47 (0.04–2.89) *p* = 0.42	0.78 (0.22–2.53) *p* = 0.68	3.29 (0.17–482.26) *p* = 0.43	1.70 (0.47–6.83) *p* = 0.42	0.95 (0.3–2.94) *p* = 0.92
Tsigai breed	4.31 (0.54–87.96) *p* = 0.21	0.19 (0.001–2.04) *p* = 0.19	**4.02 (1.64–10.89) *p* = 0.003**	**22.25 (2.65–2904.02) *p* = 0.002**	1.05 (0.20–4.88) *p* = 0.95	**5.3 (2.22–14.12) *p* < 0.001**
Sex	1.97 (0.1–37.04) *p* = 0.65	4.23 (0.21–85.84) *p* = 0.35	1.55 (0.18–13.37) *p* = 0.69	2.24 (0.12–42.48) *p* = 0.59	1.37 (0.07–25.18) *p* = 0.83	1.29 (0.15–11.08) *p* = 0.81
Age	1.06 0.75–1.35 *p* = 0.69	1.00 (0.55–1.43) *p* = 0.99	1.07 (0.89–1.24) *p* = 0.44	1.17 (0.88–1.46) *p* = 0.2	1.04 (0.78–1.29) *p* = 0.74	1.11 (0.95–1.28) *p* = 0.17
Herd size	1.00 (0.99–1.01) *p* = 0.91	1.00 (0.99–1.01) *p* = 0.96	0.99 (0.99–1.002) *p* = 0.4	0.99 (0.99–1.007) *p* = 0.95	1.00 (0.99–1.01) *p* = 0.9	0.99 (0.99–1.003) *p* = 0.52
Shearing	1.01 (0.25–4.13) *p* = 0.99	0.67 (0.07–6.53) *p* = 0.73	0.39 (0.14–1.06) *p* = 0.06	0.28 (0.03–2.3) *p* = 0.23	0.89 (0.27–2.98) *p* = 0.85	**0.4 (0.16–0.99) *p* = 0.048**
Clinical signs	1.06 (0.13–8.8) *p* = 0.95	0.89 (0.05–16.92) *p* = 0.94	2.41 (0.9–6.48) *p* = 0.08	2.81 (0.54–14.51) *p* = 0.22	0.68 (0.09–5.39) *p* = 0.71	2.46 (0.97–6.22) *p* = 0.06

^*^Reference category in the logistic regression; the ANOVA *p*-value represents the influence of breed; WNV, West Nile virus; USUV, Usutu virus; TBEV, tick-borne encephalitis virus; mosquito-borne pathogens: WNV/USUV; tick-borne pathogens: TBEV/*B. burgdorferi* s.l. Statistically significant differences are bold.

Of the other risk factors analyzed, only sheep shearing was statistically significant, with unshared sheep showing a higher probability of tick-borne infections (*p* = 0.048) (Table 4).

## 4 Discussion

Infection of sheep with various zoonotic VBD pathogens has been documented in several studies, but their use as sentinel animals needs to be evaluated. In this study, we analyzed the seroprevalence of zoonotic VBDs in sheep from WNV and TBEV endemic areas in Croatia, as well as areas with sporadic evidence of USUV infection. In addition, we tested sheep for *B. burgdorferi* s.l., the most common vector-borne bacteria in Croatia, and the possible introduction of CCHFV. Sheep in all three sampling micro-locations and on all tested farms were positive for TBEV, WNV, USUV, and *B. burgdorferi* s.l. antibodies, suggesting their possible sentinel role for selected zoonotic pathogens.

The highest seroprevalence was recorded for TBEV (9.7%). It was higher than the 0.53 and 0.42% seroprevalence in Germany and Belgium, tested by VNT and plaque reduction neutralization test, respectively (29, 30). The observed difference could be attributed to the endemic occurrence and high risk of TBEV infection in this part of Croatia (22).

Very few studies analyzing WNV seroprevalence in sheep are available for European countries. In a study conducted in Turkey, 1% of sheep tested positive for WNV neutralizing antibodies (31). A seropositivity of 2.2% was observed by VNT in the Astrakhan region of Russia (32). The WNV seroprevalence of 3.0% in Croatia is similar to the seroprevalence results in sheep from Egypt, northeast Ethiopia, and Tunisia (3.5, 3.5, and 3.2%, respectively), which are also enzootic regions for WNV (33–35). With extremely high seroprevalence in horses (36), the surveillance system in this area could be compromised, making sheep a possible alternative species for WNV surveillance in regions with high virus activity.

To the best of our knowledge, this study gives the first serological evidence of USUV infections in sheep globally. The detection of USUV-seropositive sheep in Vukovar-Srijem County is not surprising, as the first serologic evidence of human USUV infection in Croatia was confirmed in 2012 in a resident of this region (37).

Athanosiu et al. (38) confirmed a *B. burgdorferi* s.l. seroprevalence of 23.58% in sheep in Greece. Similarly, a seroprevalence study in Slovakia found the seropositivity to *B. burgdorferi* s.l. of 15.8% (1999) and 17.5% (2000) (39). In the Alto Adige-South Tyrol, Italy, the seropositivity in sheep was 14.1% (1990) (40). In the present study, seropositivity was much lower (2.7%), which is consistent with the low incidence of human borreliosis in this Croatian region (21).

All sheep were tested negative for CCHFV, which was expected since this arbovirus had not yet been confirmed in Croatia. Further investigation is necessary due to the high risk of CCHF emergence, given the recent seropositive sheep confirmation in neighbouring countries (41, 42).

The antibody detection in sheep suggests their sentinel potential for VBD pathogens. For highly prevalent pathogens such as WNV, the possible advantage of sheep in endemic areas is noteworthy. In Vukovar-Srijem County, the testing area in the present study, a high IgG seroprevalence of WNV exceeding 50% was recorded in horses during 2024 (data of the Faculty of Veterinary Medicine, University of Zagreb). This challenges the continued use of horses as sentinel animals in the upcoming transmission season and underscores the need for integrating additional species into the surveillance framework. The easy access to serum samples collected for veterinary important infectious disease surveillance, such as brucellosis, and the large population of these animals offer the advantage of including sheep in the surveillance programme for VBD pathogens.

In addition to confirming the activity of zoonotic pathogens in specific areas, surveillance with sentinel animals could provide information on epidemiological risk factors in a particular location. In this study, we confirmed that location had a significant influence on the risk of infection with VBD pathogens. This was not confirmed for WNV and mosquito-borne pathogens overall, likely due to the vector population density and the high viral activity of WNV observed in this area over the last decade. The impact of location in a small geographic area, as investigated in this study, on mosquito-borne infections could be minimized compared to tick-borne diseases, given that mosquitoes travel long distances, with a mean distance of 1.33 km (43). Even though the risk of infection from less prevalent mosquito-borne pathogens, such as USUV, in this region is location-dependent in some instances. This indicates a general and highly prevalent spread of WNV throughout the study area, as well as the circulation of USUV in some micro-foci.

Location was a significant risk factor for TBEV, *B. burgdorferi* s.l., as well as tick-borne diseases overall. The observed differences between mosquito-borne and tick-borne VBDs can be explained by the significantly different movement distances of ticks compared to mosquitoes. Slovák et al. (44) confirmed that nymphs have the highest infection rates with TBEV among tick stages. At the same time, the independent movement of nymphs is very short, and they were predominantly recaptured 2–3 meters from their release points, while almost 50% of adults were found to be more than 5 meters away, with some dispersing up to 7–8 meters. These findings confirm that all tick stages exhibit minimal autonomous movement (without involvement of the host’s movement), resulting in microlocalization of TBEV circulation.

From the other geographic and environmental risk factors for VBDs in this study, we found that the distance of the flock from the household is a significant risk factor for TBEV, *B. burgdorferi* s.l., and tick-borne diseases overall. This finding could be the result of the above-explained differences in the movement of the vectors, as well as confirmation that the dispersal of nymphs into pastures was minimal compared to the woodland (45).

In an analysis of host- and management-related risk factors for infection with VBD pathogens in sheep, we confirmed breed as an important risk factor. Due to the high correlation between breed and location, this finding requires further investigation. Sex, age, flock size, and clinical signs did not influence seroprevalence. An unexpected result was that the risk of tick-borne infection was statistically higher in flocks without sheep shearing practices. This could be a consequence of the easier attachment of tick vectors to a host when sheep were not sheared, but may also reflect broader farming practices. Regularly shared flocks are handled more frequently, increasing the likelihood of early tick infestation detection. Differences in exposure risk may also be influenced by other factors not addressed in our study, such as the level of flock management and the degree of system extensiveness, which is a limitation of the study. The role of shearing is, therefore, a risk factor likely multifactorial in origin and should be interpreted within the broader context of flock management and environmental exposure.

In conclusion, the results of this study suggested the potential of sheep as sentinels for mosquito-borne and tick-borne zoonotic diseases. The lower WNV seroprevalence in sheep compared to horses, the most commonly used sentinel species for WNV, suggests that even sheep may have advantages in endemic regions with high viral activity. In this study, to the best of our knowledge, we confirmed for the first time USUV infection in sheep, which expands the possibility of collecting epidemiological data using this species as sentinel animals. However, it should be noted that age was not identified as a significant risk factor for VBDs seropositivity. Due to cumulative exposure, older animals are often more likely to be seropositive, and their usefulness as sentinels for identifying recent pathogen circulation may be questionable. Therefore, further studies are needed to evaluate the role of sheep as sentinels for mapping microfoci of zoonotic pathogens’ activity and determining high-risk areas for public health.

The findings of this study are relevant within the “One Health” framework, which emphasizes the interdisciplinary collaboration between human, animal, and environmental health. The detected seropositivity to multiple zoonotic VBD pathogens (TBEV, WNV, USUV, *B. burgdorferi* s.l.) in sheep in various micro-locations supports the inclusion of livestock surveillance in integrated early-warning systems. By highlighting geographic and environmental risk factors such as farm location, breed susceptibility, and proximity to households, the study provides insight into the micro-epidemiology of zoonotic VBDs. These data can be used to improve risk mapping at the local level, allowing for more focused public health interventions, such as tick and mosquito control measures and public education campaigns in high-risk zones.

## Data Availability

The raw data supporting the conclusions of this article will be made available by the authors, without undue reservation.
