# Supplementary material for: Seroprevalence study for selected zoonotic vector-borne pathogens in sheep from endemic areas of Croatia
Source: Front Vet Sci. 2025 Jun 2;12:1602706. doi: 10.3389/fvets.2025.1602706 (PMC12168362; doi:10.3389/fvets.2025.1602706)
Supplement: Supplementary file 1 [file Data_Sheet_1.pdf]

Epidemiological Questionnaire – Sheep – Vector-Borne Zoonoses

**Sample ID**	
**Location**	
**Farm ID**	
**Date of sampling**	

**Flock size**<br>*(total number of animals)*	

**Breed**	☐ Ro	☐ Ts	☐ Me
	☐ Other ____________		
**Age**<br>*(years)*			
**Sex**	☐ M	☐ F	

**Clinical signs in animals**<br>(*in the past year*)	☐ CNS	☐ Res	☐ Rep	☐ GI	☐ Der	☐ Lam
	☐ Other ____________					

**Type of water bodies**	☐ R	☐ S	☐ L
**Distance to water bodies**<br>*(km)*			
**Distance to households**<br>*(km)*			
**Shearing**	☐ Y	☐ N	

**Other notes:**

Legend:

Breed: *Ro-Romanov; Me-Merinolandschaf; Ts-Tsigai;*

Sex: *M-Male; F-Female*

Clinical signs: *CNS – neurological; Res – respiratory; Rep – reproductive; GI – gastrointestinal; Der – dermatological, Lam – lameness;*

Type of water bodies: *R -river; S – stream; L - lake*
